# Positive Orientation and Feelings of Loneliness among Polish Students during the COVID-19 Pandemic

**DOI:** 10.3390/jcm13113192

**Published:** 2024-05-29

**Authors:** Ewa Kupcewicz, Kamila Rachubińska, Anna Maria Cybulska, Paweł Jastrzębski, Aleksandra Bentkowska, Elżbieta Grochans

**Affiliations:** 1 Department of Nursing, Collegium Medicum, University of Warmia and Mazury in Olsztyn, 14 C Zolnierska Street, 10-719 Olsztyn, Poland; ekupcewicz@wp.pl; 2 Department of Nursing, Pomeranian Medical University in Szczecin, 48 Zolnierska Street, 71-210 Szczecin, Poland; anna.cybulska@pum.edu.pl (A.M.C.); elzbieta.grochans@pum.edu.pl (E.G.); 3 Department of Emergency Medicine, Collegium Medicum, University of Warmia and Mazury in Olsztyn, 14 C Zolnierska Street, 10-719 Olsztyn, Poland; pawel.jastrzebski@uwm.edu.pl; 4 Hospital Emergency Department, Provincial Specialist Hospital in Olsztyn, 18 Zolnierska Street, 10-719 Olsztyn, Poland; a.bentkowska.kruszwicka@gmail.com

**Keywords:** COVID-19, loneliness, positive orientation, students

## Abstract

**Background/Objectives**: The COVID-19 pandemic was a time of limited direct contact with other people. The aim of this study was to determine the role of positive orientation and sociodemographic variables in the feelings of global, social and emotional loneliness and to seek predictors for loneliness among students during the COVID-19 pandemic. **Methods**: The study was conducted on a group of 798 students at the University of Warmia and Mazury in Olsztyn (Poland) between January and March 2022. The empirical data were gathered with the following research tools: the Positive Orientation Scale and the De Jong Gierveld Loneliness Scale—DJGLS. **Results**: The study found a correlation (r = −0.370; *p* < 0.001) between positive orientation and emotional loneliness. The level of loneliness was significantly higher in students who greatly reduced their social contact, compared to those who reduced them moderately (*p* < 0.001). The intensity of social loneliness among women was lower (*p* < 0.001) than among men. Those who lived with their families or with a close companion felt less intense emotional loneliness (*p* < 0.001) than those who lived alone. **Conclusions**: A positive orientation, which manifests itself in a favourable opinion about oneself and in attaching weight to positive aspects of life, was found to be the main determinant of the feelings of global and emotional loneliness. However, it did not prove to be a predictor of social loneliness in the group of students under study.

## 1. Introduction

Restrictions imposed to contain the spread of SARS-CoV-2 in 2020 resulted in changes in the higher education system and in a transition from traditional face-to-face teaching–learning methods to online education [1]. Together with limited social contact, these changes contributed to increased difficulties in learning among students of various majors—medical, technical and humanistic [2]. Moreover, students had to cope with restrictions concerning their everyday lives, social relations, free time and learning [3,4,5]. These changes, in turn, brought about an increase in feelings of loneliness, uncertainty and isolation, and difficulties in staying in touch with friends, relatives, peers and academic teachers [6]. According to the findings of many studies, students were susceptible to mental health issues with a growing tendency during the COVID-19 pandemic [7,8].

Because of the specific nature of the medical and veterinary professions, individuals practicing them, including students, must be psychologically resilient, empathetic to pain and suffering, compassionate and eager to provide assistance. Therefore, it is worth noting the importance of psychological factors and learning about their functions in achieving success in one’s profession and in life. Positive orientation is one of these important factors. The concept of positive orientation arose from the generalisation of the results of empirical studies—self-esteem, life satisfaction and optimism were recursively correlated with each other and formed a single factor in the results of factor analyses. These analyses allowed the authors to formulate the hypothesis that there is a common underlying latent variable. Positive orientation is the underlying tendency to notice and attach importance to positive aspects of life, experiences and oneself. It is largely responsible for adaptive functioning, as it signifies a natural inclination towards a favourable evaluation of oneself, high satisfaction with life and a high evaluation of one’s chances of achieving one’s goals, which translates into a commitment to life pursuits and a high evaluation of one’s quality of life [9,10]. Examining its impact on the level of loneliness will provide knowledge of psychological strength, which will allow for the improvement of future health workers and veterinarians in the professional and family environment. A tendency to seek positive aspects in crisis situations is a universal human experience [11]. The available study reports show positive orientation with respect to the crisis caused by the pandemic [12]. Positive orientation was measured using three separate scales: self-esteem, optimism and life satisfaction. Based on the results, a factor score was calculated, which could only be obtained after a factor analysis on the overall results of the three scales. The scales themselves had a relatively large number of statements (especially taking into account the fact that, in the end, only one indicator of the latent variable was derived from them), and the procedure for the calculation of the factor score required appropriate skills. Therefore, a short scale that was an extract of the three initial components was proposed instead. The Positivity Orientation Scale (abbreviated as P-Scale), which greatly simplified and shortened the study and had good psychometric properties, was used in the study [13].

Positive orientation is part of positive psychology, which analyses and seeks resources that can help an individual not only to face hardships but also to overcome them. The concept of positive orientation is a result of the generalisation of study findings concerning self-esteem, satisfaction with life and optimism, which are correlated with many factors. These correlations are indicative of individual optimum functioning and well-being. Positive orientation is the reverse of Beck’s cognitive triad, which comprises a negative opinion about oneself, the world and the future. Like the features of the Great Five, they are largely hereditary [14]. According to Caprara [15], positive orientation is an individual’s basic predisposition to note and attach weight to positive aspects of life, experience and oneself. It is largely responsible for the adaptive functioning of an individual, as it is a natural tendency to hold a favourable opinion about oneself, to be satisfied with life and to perceive the likelihood of the fulfilment of one’s goals as good, which contributes to involvement in pursuing life goals and a high opinion about one’s quality of life. This allows an individual to cope in life despite failures, hardships and the prospect of death. A predisposition to believe that one is a respectable person, that life is worth living and that the future is promising allows one to bear the knowledge of one’s limitations and to cope with the difficulties, hardships and losses in one’s life [16].

Medicine and veterinary students prepare to fulfil their duties towards other human beings, both in illness and in health. For this reason, their mental well-being is important not only to themselves but also for the quality of the healthcare that they provide [17]. According to a literature review, the issue of positive orientation is rarely dealt with in the context of coping with emotions, feeling lonely and students’ academic lives [18]. Therefore, it was accepted, based on theoretical assumptions and empirical data, that this study would provide a significant contribution to the literature.

The research conducted so far has focused on the consequences of the restrictions imposed on society to control the spread of the SARS-CoV-2 pandemic. One of the consequences identified is loneliness [19,20]. Surprisingly, nearly half of the population aged 18–24 years reported feeling a higher level of loneliness than elderly people during their isolation [21].

Loneliness is a multi-faceted, multi-cause and multi-dimensional phenomenon. A feeling of loneliness is defined as a negative mental response to a discrepancy between expectations in the social sphere and the actually occurring relations. It is caused by a lack of social relations or a sufficient level of intimacy in existing ones. Loneliness is also defined as the reverse of social support [20]. The literature on the subject identifies emotional and social loneliness. There are considerable differences between them. According to many scholars, emotional loneliness concerns the lack of the point of attachment, which is associated with a feeling of isolation. On the other hand, social isolation manifests itself as the lack of a network of social support and the lack of a circle of close people who provide a space to develop a sense of belonging and be part of a community [22]. There are many tools to measure feelings of loneliness. According to Pinquart and Sörensen [23], the Jenny de Jong Gierveld Loneliness Scale (DJGLS) is the most commonly used scale in research practice. The DJGLS measures the two aforementioned dimensions of loneliness (social and emotional), which are generalised to a higher-order factor of overall feelings of loneliness (two-factor structure).

According to many scholars, loneliness has created a new pandemic. This state affects a third of the population in developed, industrialised countries. Moreover, a growing tendency is observable. The problem affects 20 to 48% of the young and young adult population. These people are also found to report high levels more frequently. It is believed that this population is at the greatest risk of an increase in feelings of loneliness [20,24,25].

During and after the COVID-19 pandemic, loneliness became an issue that was dealt with increasingly often in the social sciences. This is mainly a consequence of the measures aimed at restricting contact in order to contain the spread of the COVID-19 pandemic, which resulted in a considerable reduction in social contact. Although the lack or restriction of social contact does not always lead to loneliness, it is regarded as a risk factor. Many papers have recently been published about loneliness during the pandemic. The topic is popular, especially among authors who deal with quality of life. The risk factors for loneliness have been examined. A study conducted in the Netherlands [26] showed an increase in the level of loneliness between 2019 and 2020. A particularly distinct growth in emotional loneliness was observed in the Dutch study. Having a partner and a high level of physical functioning were mentioned as protective factors. The COVID-19 Psychological Wellbeing Study in the UK showed that age (young people are more susceptible), separation and divorce were among the risk factors. On the other hand, social support and being in a relationship are protective factors [27].

A review of the literature showed that there were associations between loneliness and a sense of meaning in life. Loneliness, defined as subjective dissatisfaction with the quality of one’s interpersonal relationships, was negatively associated with the meaning domain of well-being. Moreover, loneliness was found to be a better predictor of a sense of meaning in life than depression, pessimism and a negative mood. Studies have shown that loneliness may be a predictor of positive life orientation [28,29]. Cacioppo et al. [30] found that loneliness reduced respondents’ levels of self-esteem and optimism. Loneliness can undermine self-esteem, a positive assessment of one’s life and an optimistic view of one’s future, thus making it difficult to perceive the meaning of one’s existence. Academic study can be a challenge for many students, which has been shown in research demonstrating a high frequency of psychosocial disorders among students [31,32]. This mainly concerns medicine and veterinary students, who are required to show empathy and a willingness to help other people or—in the case of veterinary students—animals [33]. The COVID-19 pandemic was a time of limited direct contact with other people. This period was particularly difficult for people accustomed to active participation in social life, especially students. It is possible that such people experienced a growing feeling of loneliness, and the legal regulations concerning the rules to be followed during the pandemic and distance learning had particularly severe impacts on students, necessitating a change in lifestyle and making their feelings of loneliness more acute [34].

The aim of this study was to determine the role of positive orientation and sociodemographic variables in the feelings of global, social and emotional loneliness and to seek predictors for loneliness among students during the COVID-19 pandemic.

## 2. Materials and Methods

### 2.1. Settings and Design

The study sample included 798 students at the University of Warmia and Mazury in Olsztyn, Poland in the following majors: nursing, midwifery, emergency medicine, dietetics and veterinary medicine. Having obtained the required consent, the researchers conducted the survey in direct contact with the students, while maintaining the required sanitary regime, during the period from January to March 2022. The provision of informed consent to participate in the study, the absence of diagnosed physical or mental illnesses and being under 30 years old were the inclusion criteria. Those who failed to give such consent were excluded from the study. The group was selected at random. No randomisation tool was applied. The study was carried out in groups of approximately a dozen students. It was anonymous, participation was voluntary, and the participants were not paid. The participants were informed about the study objective and had the opportunity to ask questions and receive answers. The students could withdraw from the study at any time, without providing a reason. It took approximately 15 min to complete the questionnaire. A total of 850 questionnaire sets were distributed to the students. After the data’s completeness was verified, 798 were taken for the statistical analysis (return rate 93.88%). All ethical norms for the performance of scientific studies were followed. This study was part of a larger research project for which a favourable opinion (No. 3/2021, approved on 15 June 2021) was received from the Senate Scientific Research Ethics Committee at Olsztyn University in Olsztyn, Poland [35]. The research project aims to assess the psychosocial functioning of students during the COVID-19 pandemic, as well as the long-term effects of the pandemic that may affect the bio-psycho-social state among students. The research also aims to identify the need for institutional support for students, as well as psychological support if needed, especially in situations where, among other factors, interpersonal contacts are limited.

### 2.2. Research Instruments

A diagnostic survey method was applied, and the following research tools were used to collect the data:

- a proprietary questionnaire, which included questions about sociodemographic data, i.e., gender, age, place of residence, year of study, number of meals per day, number of hours of work with a computer and extent to which social contacts and physical exercise were reduced;
- the Positive Orientation Scale (the Positivity Scale—P-Scale), developed by Caprar et al. in the Polish version [36];
- the Loneliness Measurement Scale (De Jong Gierveld Loneliness Scale—DJGLS), developed by J. de Jong-Gierveld and F. Kamphuis, in a Polish adaptation by Grygiel et al. [37].

#### 2.2.1. The Positivity Scale (P-Scale)

The positivity scale is used to measure an individual’s tendency to notice and appreciate the positive aspects of life, experiences and oneself. It consists of eight diagnostic statements, and participants are asked to indicate their level of agreement on a 5-point scale, ranging from 1 (I definitely disagree) to 5 (I definitely agree). One statement, No. 4, utilizes reversed scoring. The total score reflects the individual’s positive orientation, with raw scores ranging from 8 to 40 points. These raw scores are then converted to standardised units using the Sten scale for interpretation. Scores between 1 and 4 Sten are considered low, 5 and 6 Sten are average and 7 to 10 Sten are high. The Polish version of the P-scale demonstrates good psychometric properties, with internal consistency (Cronbach’s alpha) ranging from 0.77 to 0.84. The P-Scale is mainly applied in scientific research, aiming to determine which aspects of human functioning are linked to positive orientation and its adaptive significance. The results of research using the Polish version of the Positive Orientation Scale indicate that it can be considered a reliable and accurate tool for the measurement of positive orientation. The P-Scale is mainly applied in scientific research aiming to determine the connection of positive orientation with individual aspects of human functioning and the adaptive significance of positive orientation. The short and easy-to-use method facilitates research into this construct. It is also possible that the scale could be used in clinical research, given its cost-effectiveness and correlations with feelings of hopelessness [36].

#### 2.2.2. De Jong Gierveld Loneliness Scale (DJGLS)

The scale for loneliness measurement consists of 11 statements, which include six negative statements on the lack of satisfaction with social contacts (social loneliness) and five positive statements measuring satisfaction with interpersonal relations (emotional loneliness). The level of acceptance of each statement is marked on a five-point Likert scale: 1—definitely yes, 2—yes, 3—neither yes nor no, 4—no, 5—absolutely not. The higher the total score for a respondent, the higher their sense of loneliness. The scale has good psychometric properties. The internal stability index (Cronbach’s alpha) is high at 0.89, with inter-item correlation r = 0.42 and Loevinger’s H coefficient of homogeneousness = 0.47. The translation process followed the latest guidelines for the cross-cultural adaptation of questionnaires and was tested by analysing differential item functioning (DIF) using the Poly-SIBTEST method and bilingual groups. The study found no differences in item performance between the final Polish and English versions and confirmed previous findings indicating that the DJGLS measures two dimensions of loneliness (social and emotional) that are generalised to a higher-order factor of the overall feeling of loneliness (two-factor structure). Studies have shown that the tool has a satisfactory level of accuracy: its correlation with the UCLA Loneliness Scale r = 0.82; with the Rosenberg Self-Esteem Scale r = −0.56; and with the Beck Depression Inventory r = 0.46 (all *p* < 0.01). The Polish adaptation of the DJGLS presents a two-factor structure with a good level of internal consistency, homogeneity and construct validity [37].

### 2.3. Statistical Analysis

The statistical analysis was performed using the STATISTICA v.13.3 software (TIBCO, Palo Alto, CA, USA). The variables were described using descriptive statistics methods, including the arithmetic mean (M), median (ME), standard deviation (SD) and minimum–maximum (Min.–Max.). The shape and symmetry of the distribution were determined using skewness and kurtosis. A 95% confidence interval for the mean was also established. The impact of sociodemographic variables on the intensity of global, social and emotional loneliness was assessed using intergroup one-way analysis of variance with the Fisher F test. Specific analyses were conducted using the post hoc test (LSD). The Pearson correlation (r) was used to examine the significance of the correlations between the variables under analysis. The strength of the correlations was interpreted based on Guilford’s classification: |r| = 0—no correlation, 0.0 < |r| ≤ 0.1—slight correlation, 0.1 < |r| ≤ 0.3—weak correlation, 0.3 < |r| ≤ 0.5—average correlation, 0.5 < |r| ≤ 0.7—high correlation, 0.7 < |r| ≤ 0.9—very high correlation, 0.9 < |r| < 1.0—nearly full correlation, |r| = 1—full correlation [4]. Multiple regression analysis was conducted to identify loneliness predictors. A significance level of *p* < 0.5 was adopted.

## 3. Results

### 3.1. Participants

Altogether, 798 students from the University of Warmia and Mazury in Olsztyn participated in the study, including 684 women (85.93%) and 112 men (14.07%). The mean age of the participants was 20.74 years (SD = 1.70). The majority of the respondents (n = 380; 47.74%) lived with their families or with a close companion. There were 337 (42.34%) first-year students, 197 (24.75%) second-year students and 262 (32.91%) third-year students. Nearly all of the students were satisfied with their health status. They spent 5.81 (SD = 2.66) hours a day on average working on a computer. A large portion (70%, n = 571) of the respondents had 3–4 meals a day, but usually not at the same time every day. Over 90% of them reduced their social contact because of the COVID-19 pandemic to a medium or considerable extent. Nearly two thirds reduced their physical exercise to various extents (19.47%—slightly, 23.24%—to an average extent, 23.99%—considerably). When choosing a form of physical exercise, they usually preferred walking or jogging (n = 466; 58.54%) and cycling (n = 159; 19.97%).

### 3.2. Analysis of Variables

The statistical analysis determined the overall positive orientation index in the study sample to be 26.25 points (SD = 6.14) on a scale from 8 to 40. The global loneliness index was determined to be 27.08 points (SD = 6.80) on a scale from 11 to 55. The individual categories of loneliness had the following mean values: social loneliness—15.48 (SD = 5.32), emotional loneliness—11.60 (SD = 4.29). The analysis results are summarised in Table 1.

The overall positive orientation index was subsequently converted into standardised units during further analyses. These units were interpreted according to the properties of the Sten scale. It was found that the results for over half of the students (54.02%) ranged from 1 to 4 Sten, which was indicative of a low positive orientation level. Results between 7 and 10 Sten, indicating a high positive orientation intensity, were noted for only 17.21% of the participants, whereas average results (5–6 sten) were noted for 28.77% of the respondents.

### 3.3. Analysis of Relationship between Positive Orientation and Global Loneliness and in Components of Social and Emotional Loneliness among Students under Study—Pearson Correlation Coefficient

**Figure 1:**
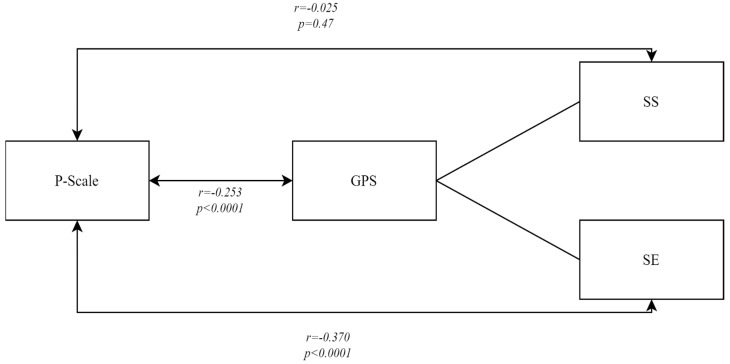
The nature and intensity of the relationship between positive orientation and global, social and emotional loneliness—Pearson correlation coefficient^®^.

An analysis of the links between positive orientation and the loneliness experienced by students during the COVID-19 pandemic revealed a significant relationship between positive orientation and global and emotional loneliness. A weak negative correlation was shown to exist between positive orientation and global loneliness: r = −0.253; *p* < 0.0001. This means that individuals with low scores in the measurement of positive orientation felt global loneliness more intensely. A specific analysis involved an attempt to examine a link between positive orientation and social and emotional loneliness. No significant relationship was found between positive orientation and social loneliness (r = −0.025; *p* = 0.47). The correlation coefficient between positive orientation and emotional loneliness was r = −0.370; *p* < 0.001, showing a significant, negative (on an average level) relationship between the variables under study. This means that individuals with a low level of positive orientation experience emotional loneliness more intensely (Figure 1).

### 3.4. Analysis of Impact of Sociodemographic and Lifestyle-Related Variables on Intensity of Global, Social and Emotional Loneliness

Subsequently, the impact of sociodemographic and lifestyle-related variables on the intensity of global, social and emotional loneliness was analysed. An intergroup one-way analysis of variance with the Fisher F test was used to demonstrate that two variables played a significant role in the intensity of global loneliness. One of them was the number of meals per day (F = 3.94; *p* < 0.008), and the other was associated with a reduction in social contact during the COVID-19 pandemic (F = 3.51; *p* < 0.01). Specific analyses with the post hoc test (LSD) showed the intensity of global loneliness in students who had only one or two meals a day to be significantly higher (*p* < 0.001) than in those students who had meals three or more times a day. The intensity of global loneliness in students who reduced their social contact to a considerable extent was significantly higher than in those who reduced such contact to an average extent (*p* < 0.001).

When seeking the factors with a significant impact on the intensity of social loneliness, it was found that this loneliness was differentiated significantly only by gender among the students (F = 4.12; *p* < 0.04). Specific analyses with the post hoc test (LSD) showed that the intensity of social loneliness among women was significantly lower (*p* < 0.001) than among men.

Further analyses showed that the factors with a significant impact on the intensity of emotional loneliness included two variables associated with the student’s place of residence during the COVID-19 pandemic (F = 12.43; *p* < 0.0004) and the number of meals per day (F = 3.22; *p* < 0.02). Specific analyses with the post hoc test (LSD) showed that those who lived with their families or with a close companion felt less intense emotional loneliness (*p* < 0.001) than those who lived alone. The intensity of emotional loneliness in students who had only one or two meals a day was significantly higher (*p* < 0.001) than in those who had three or more meals a day. Detailed data are provided in Table 2.

### 3.5. Predictors of Loneliness

The last stage of the statistical analyses involved seeking predictors for global, social and emotional loneliness. The model of standard multiple regression, performed for global, social and emotional loneliness (dependent variables), also included sociodemographic and lifestyle-related variables and positive orientation (independent variables).

An analysis of the results in Table 3 shows that only positive orientation proved to be a predictor of global loneliness in the group of Polish students, whereas the sociodemographic and lifestyle-related variables ultimately did not enter the regression model. The regression model explained only 6% of the results’ variability, and the regression coefficient for positive orientation as a predictor was negative (ßeta = −0.24; R^2^ = 0.067), indicative of a negative correlation. This means that the higher the positive orientation level, the lower the global loneliness intensity.

When seeking predictors of social loneliness, it was shown that one sociodemographic variable (gender) was included in the regression model, and it explained 1% of the results’ variability, which meant that the predictability of the variable was slight. Positive orientation had predictive power of 14% for emotional loneliness. The regression coefficient was negative (ßeta = −0.36; R^2^ = 0.145), which is indicative of a negative correlation. Another variable—the place and form of students’ residence during the COVID-19 pandemic—explained 1% of the results’ variability.

To summarise the regression results, positive orientation, which manifests itself in a favourable opinion about oneself and in attaching weight to the positive aspects of one’s life, was found to be a determinant of the feelings of global and emotional loneliness. However, it did not prove to be a predictor of social loneliness in the group of students under study.

## 4. Discussion

Loneliness was perceived as a more significant issue during the SARS-CoV-2 pandemic [31]. An average of 8–10% of the population experience loneliness at a level that makes everyday life and psychosocial functioning difficult. In addition, a growing tendency has been observed in some studies [19,21]. This phenomenon is most prevalent among young adults aged 18–25, corresponding to their university years [31]. This study determined the global loneliness index to be 27.08 points (SD = 6.80) on a scale from 11 to 55, while that of social loneliness was 15.48 (SD = 5.32) and that of emotional loneliness was 11.60 (SD = 4.29). The intensity of social loneliness among women was found to be lower than that among men.

The findings of a study conducted by Cacioppo et al. among young adults and adolescents showed loneliness to be experienced by 20–48% of the subjects. These people were also found to report high levels more frequently. This may be the highest value among all of the population groups, and it may be the group most threatened by increasing loneliness [38,39,40]. A study conducted by Diehl et al. showed that emotional loneliness was more prevalent than social loneliness among students. Both variables were positively correlated with a sense of depression and anxiety [39].

A significant role in the assessment of loneliness among students during the COVID-19 pandemic was played by the reduction in contact [40]. This study has shown that over 90% of them reduced their social contact because of the COVID-19 pandemic to a medium or considerable extent, and nearly two thirds of the respondents reduced their physical exercise to various extents. It was shown that the intensity of global loneliness was higher in students who reduced their social contact to a considerable extent, compared with those who did so to a lesser extent. Those who lived with their families or with a close companion felt less intense emotional loneliness than those who lived alone. The findings of a study conducted by Labrague et al. among medical students were similar [41]. They demonstrated a high level of loneliness among all students during the pandemic. Resilience and social support were identified as factors that protect against loneliness. Similar findings were presented by many authors conducting studies on groups of students. The pandemic created isolation and loneliness for many people. The number of people who died by suicide increased in many countries. A new ministry was established in Japan to deal with loneliness and take measures against this phenomenon. The threat to public health caused by loneliness during the pandemic was the object of an extensive study conducted in Canada [42,43]. According to its findings, the probability of loneliness is higher in women than in men, which is similar to the findings of this study.

Noticeably, online interactions, as a temporary solution during the pandemic, facilitated social contact and decreased feelings of loneliness. However, it was demonstrated that following this strategy—limiting contact to online interactions—for a long time considerably lowers one’s sense of belonging. Scientists claim that a response to negative circumstances—such as the pandemic—results in the excessive use of this medium [44]. A 2021 study that analysed the links between loneliness, the problematic use of the Internet and the COVID-19 pandemic showed an increase in feelings of loneliness that was particularly noticeable in women and young people. These two populations were more susceptible to negative moods. This observation may be responsible for the high levels of loneliness, as shown in this study, where the majority of the respondents were women aged 18–25 years [44].

The available study findings [41] indicate that people attempted to adapt to the situation during the pandemic and reduce their levels of discomfort by using the resources at their disposal. The imposed restriction of contact with other people, especially personal contact, could provoke a range of emotions, such as anger, anxiety and loneliness. Contacts with other people, or the ability to communicate, are important elements of social roles, and they promote development and well-being [40].

The mean score for positive orientation as measured in this study was 26.25 points and was lower than in the normalisation sample (29.19 points) [45], as well as in an international study among students in Spain (M = 29.20) and Slovakia (M = 29.22) [18] and in a multi-centre study in Poland, also conducted among students of medical majors (26.87 points) [46]. The findings of this study show that more than half of the respondents had scores indicative of low levels of positive orientation. On the other hand, scores indicative of high positive orientation were noted only in 17.21% of the respondents. A study conducted by Kupcewicz et al. [47] among nursing students during the SARS-CoV-2 pandemic also showed that the level of positive orientation was low in over half of the students. This may result in more intense feelings of loneliness, unfavourable self-esteem, lower quality of life and a poor opinion of one’s likelihood of achieving one’s personal goals, as well as a negative perception of one’s ability to cope with difficulties [47].

Positive orientation is a resource that generates positive emotions. Fredrickson stressed their role in restoring balance after adverse emotional events [48]. The authors of the co-agitational model of healthy coping demonstrate that focusing on positive emotions is insufficient. They claim that an ability to activate positive and negative emotions simultaneously and tolerate behavioural ambivalence increases the likelihood of skilful overworking and overcoming major life stresses [24]. Personal resources are perceived mainly as factors that allow one to cope with stressful situations, as well as those that counteract the generation of negative consequences of such situations. A relationship among one’s personal resources and the occurrence of positive changes is consistent with the assumptions made by the developers of the concept of post-traumatic growth, who attribute significant importance to the resources that an individual has before being exposed to a traumatic event—in this case, the SARS-CoV-2 pandemic [49].

The findings of the current study show that students with a low level of positive orientation felt global and emotional loneliness more intensely. Positive orientation manifests itself in a favourable opinion about oneself, and attaching weight to positive aspects of life was found to be a determinant of the feelings of global and emotional loneliness. However, it did not prove to be a predictor of social loneliness in the group of students under study. The findings of earlier studies provide the basis for a link between loneliness and positive orientation. Cacioppo et al. [24] discovered that the intensity of loneliness resulted in a decrease in self-esteem and optimism. Moreover, if one regards positive orientation as the opposite of the depressive cognitive triad, these findings are consistent with both cross-sectional and longitudinal studies showing that loneliness is a predictor of depressive symptoms [50,51,52,53]. Loneliness can be linked to a strong feeling of uncertainty. This feeling is associated with triggering a mechanism of survival, which is characterised by increased sensitivity to social threats and low self-esteem. This mechanism results in forecasting one’s future in terms of a threat and perceiving other people’s intentions as causing anxiety [24,54]. Focusing on social threats hinders a focus on long-term objectives, which are extremely important in developing one’s sense of life, particularly for students or those making changes in their professional lives [28]. This means that further studies of this construct are necessary, as positive orientation can be described as a personal resource that optimises the ways in which others function. Therefore, positive orientation should be reinforced as it is linked with a feeling of loneliness [29,55].

### Practical Limitations/Implications

These considerations concerning the role of positive orientation and sociodemographic data in the feeling of loneliness and its predictors among students during the COVID-19 pandemic identify certain limitations and implications for professional practice. Effective measures are necessary to modify all tested predictors of loneliness. Such measures can not only be effective in counteracting loneliness but also in restoring the optimum positive orientation in students. Therefore, it is important to implement institutional support for students and, if needed, psychological assistance, especially when interpersonal contacts are restricted. The main strengths of this study included taking into consideration important and reliable psychometric tools. Therefore, it considerably enriches the existing literature on positive orientation and loneliness during the SARS-CoV-2 pandemic and provides further insights into the nature of addiction to work and its links with health and mental well-being. This is one of the first such extensive studies conducted during the COVID-19 pandemic among medicine and veterinary students. Its other strengths include the large number of participants and the fact that they were students of both medical and veterinary majors, who constituted a representative sample for the Warmińsko-Mazurskie Voivodship. However, the study has its limitations, especially due to the lack of data on the intensity of the loneliness felt by students, the predictors of loneliness (e.g., the number of meals, the number of hours spent working on a computer and a reduction in physical exercise) and the level of positive orientation before the pandemic, which could be important for the current levels of the variables under analysis. Another limitation is the uneven distribution of the sample with respect to gender. Since medical professions are dominated by women, these results should not be generalised to include the male population. The sample was mainly composed of students from a university in Poland, which may limit the generalisation of the research results to other geographical locations, cultures or types of universities, as the research results may differ in more diverse samples or in different cultural or educational environments. The study’s cross-sectional nature limits the ability to draw causal negotiations from the data, suggesting the need for long-term research to track changes over time in longitude and positive orientation. Another limitation is that the self-reported questionnaires may have introduced social desirability biases. Despite its limitations, this study has provided important conclusions and can offer a starting point for more extensive research on these issues in the future.

## 5. Conclusions

The COVID-19 pandemic brought about important changes in the education of university students and in their general lifestyles. Students experienced distance learning and the restriction of interpersonal contact. The majority of the study participants reduced their social contacts and physical activity because of the COVID-19 pandemic to a considerable extent.

This study expands the knowledge of the role of positive orientation and sociodemographic variables in the feelings of loneliness among students during the COVID-19 pandemic. More than half of the students under study showed a low level of positive orientation. Positive orientation, which manifests itself in a favourable opinion about oneself and attaches weight to positive aspects of life, was found to be a determinant of the feelings of global and emotional loneliness. However, it did not prove to be a predictor of social loneliness in the group of students under study. The intensity of social loneliness among women proved to be lower than that among men. The predictors of loneliness included a reduction in social contact, living without close companions and being female. These considerations can offer a starting point for more extensive research on these issues and can help decision-makers, educational institutions and mental health professionals to develop strategies to improve student well-being during similar crises.

## Figures and Tables

**Table 1:** Descriptive statistics.

Variable		n = 798						
		M	95% CI	Me	Min.–Max.	SD	Skewness	Kurtosis
P-Scale		26.25	25.83–26.68	27	8–40	6.14	−0.32	−0.01
GPS		27.08	26.61–27.55	27	11–50	6.80	0.26	−0.22
Components of loneliness	SS	15.48	15.11–15.85	15	6–30	5.32	0.31	−0.26
	SE	11.60	11.31–11.90	11	5–25	4.29	0.49	−0.04

N—sample size, M—arithmetic mean, 95% CI—confidence interval of the mean, Me—median, Min.—minimum, Max.—maximum, SD—standard deviation, P-Scale—positive orientation, SS—social loneliness, SE—emotional loneliness, GPS—global loneliness.

**Table 2:** The test results for the significance of the impact of sociodemographic and lifestyle-related variables and on the intensity of global, social and emotional loneliness.

Variable			GPS					SS					SE				
			M	SD	F	*p*-Value	Post Hoc (NIR)	M	SD	F	*p*-Value	Post Hoc (NIR)	M	SD	F	*p*-Value	Post Hoc (NIR)
Gender	woman	A	26.90	6.81	3.35	0.07	-	15.32	5.32	4.12	0.04	A < B	11.58	4.36	0.15	0.69	-
	man	B	28.17	6.67				16.42	5.21				11.75	3.86			
Year of study	first	A	27.06	6.82	0.002	0.99	-	15.37	5.33	0.78	0.46	-	11.69	4.21	1.20	0.30	-
	second	B	27.11	6.86				15.22	5.07				11.88	4.30			
	third	C	27.08	6.76				15.80	5.48				11.29	4.38			
Age (years)	≤20	A	27.05	6.67	1.04	0.15	-	15.34	5.27	1.12	0.39	-	11.72	4.19	0.46	0.62	-
	21	B	27.64	6.98				15.98	5.14				11.65	4.30			
	≥22	C	26.66	6.88				15.29	5.54				11.37	4.45			
Place and form of residence	with family/close companion	A	26.77	6.93	1.5	0.22	-	15.72	5.46	1.57	0.2	-	11.05	4.21	12.43	0.0004	A < B
	alone	B	27.36	6.68				15.25	5.18				12.11	4.30			
Number of hours spent working on a computer	≤3 h	A	26.76	6.72	1.17	0.31	-	15.37	4.92	1.7	0.18	-	11.38	4.34	0.28	0.75	-
	4–7	B	27.44	6.66				15.79	5.29				11.65	4.23			
	≥8 h	C	26.66	7.10				14.98	5.63				11.69	4.37			
Number of meals	1–2	A	28.56	7.84	3.94	0.008	A > B. C. D	16.08	5.84	1.2	0.30	-	12.48	4.98	3.22	0.02	A > B, C, D
	3	B	27.09	6.50				15.53	5.20				11.56	4.02			
	4	C	26.52	6.25				15.19	5.03				11.33	4.02			
	≥5	D	25.74	7.13				14.88	5.58				10.86	4.56			
Reduction in physical exercise during the pandemic	no	A	27.37	6.92	0.71	0.39	-	15.65	5.08	0.4	0.52	-	11.72	4.51	0.31	0.57	-
	yes, to a small extent	B	26.63	6.54				15.10	5.26				11.53	3.94			
	yes, to a medium extent	C	27.08	6.66				15.49	5.51				11.59	4.28			
	yes, to a considerable extent	D	27.05	7.00				15.53	5.51				11.51	4.28			
Reduction in social contact during the pandemic	to a small extent	A	27.38	7.74	3.51	0.01	B < C	16.08	5.90	2.38	0.06	-	11.30	4.87	1.53	0.2	-
	average/medium	B	26.47	6.40				15.11	4.91				11.36	4.18			
	considerable	C	28.17	7.22				16.11	5.86				12.06	4.25			
	very large	D	26.57	6.49				14.87	4.93				11.70	4.62			

M—arithmetic mean, SD—standard deviation, GPS—global loneliness, SS—social loneliness, SE—emotional loneliness.

**Table 3:** Summary of regression—predictors of global, social and emotional loneliness.

Variable		R^2^	ßeta	ß	t	*p*-Value
GPS	Constant value			34.96	20.83	0.0000
	P-Scale	0.06	−0.24	−0.27	−6.81	0.0000
	R = 0.26; R^2^ = 0.072.; corrected R^2^ = 0.067					
SS	Constant value			16.64	14.11	0.0000
	Gender	0.01	0.07	1.09	2.02	0.04
	R = 0.11; R^2^ = 0.01; corrected R^2^ = 0.008					
SE	Constant value			16.46	15.45	0.0000
	P-Scale	0.14	−0.36	−0.25	−10.94	0.0000
	Place and form of residence	0.01	0.10	0.84	2.97	0.003
	R = 0.38; R^2^ = 0.148; corrected R^2^ = 0.145					

GPS—global loneliness, SS—social loneliness, SE—emotional loneliness.

## Data Availability

The data presented in this study are available on request from the first author.
